# Predictors of Early Mortality in Cancer-Associated Thrombosis: Analysis of the RIETE Database

**DOI:** 10.1055/s-0038-1642022

**Published:** 2018-04-19

**Authors:** Alfonso J. Tafur, Harry Fuentes, Joseph A. Caprini, Agustina Rivas, F. Uresandi, Rita Duce, Raquel Lopez-Reyes, Adriana Visona, Adel Merah, Manuel Monreal

**Affiliations:** 1 NorthShore University HealthSystem, Evanston, Illinois, United States; 2 John Stroger Cook County Hospital, Chicago, Illinois, United States; 3 Hospital Universitario Araba, Álava, Spain; 4 Hospital de Cruces, Barakaldo, Vizcaya, Spain; 5 Ospedale Galliera, Genova, Italy; 6 Hospital Universitari i Politècnic La Fe, Valencia, Spain; 7 Ospedale Castelfranco Veneto, Castelfranco Veneto, Italy; 8 Université Jean-Monnet, Service de Medecine Vasculaire et Therapeutique, CHU de Saint Etienne, Saint-Etienne, France; 9 Hospital Universitario Germans Trias i Pujol de Badalona, Universidad Católica de Murcia, Barcelona, Spain

**Keywords:** mortality, cancer, venous thromboembolism

## Abstract

Cancer-associated thrombosis (CT) carries a high, heterogeneous, and poorly predicted likelihood of mortality. Thus, we aimed to define predictors of 30-day mortality in 10,025 patients with CT. In a randomly selected derivation cohort, we used recursive partitioning analysis to detect variables that select for a risk of mortality within 30 days. In a validation cohort, we evaluated our results using Cochran–Armitage test. The most common types of cancer were lung (16%), breast (14%), and colorectal (14%); median age was 69 years (range, 14–101); most had metastatic disease (63%); 13% of patients died within 30 days. In the derivation cohort ( *n* = 6,660), a white blood cell (WBC) count in the highest quartile predicted early mortality (odds ratio, 7.8; 95% confidence interval [CI], 4.6–13.1); and the presence of metastatic disease, pulmonary embolism (PE), and immobility defined the risk of those with normal WBC count. We defined death risk according four sequential questions: (1) Does the patient have an elevated WBC count? (Yes, group D). (2) If no, does the patient have metastasis? (No, group A). (3) If yes, is the patient immobile? (Yes, group D). (4) If no, does the patient have a PE? (Yes, group C; no, group B). In the validation cohort ( *n* = 3,365), the 30-day risk of death was 2.9% in group A (95% CI, 1.9–4.3), compared with 25% in group D (95% CI, 22.5–27.5), and there was a rate escalation between groups ( *p* for trend < 0.01). In conclusion, with four sequential questions, the risk of death in CT can be easily stratified. An elevated WBC count at baseline predicted 30-day mortality better than metastases, PE, or immobility.

## Introduction

Cancer-associated thrombosis (CT) is a frequent, potentially preventable complication of cancer treatment, which is independently associated with higher rate of mortality. 1 2 Primary thromboprophylaxis strategies using low-molecular-weight heparin (LMWH) have been tested in randomized trials and led to a defined reduction in the likelihood of venous thromboembolism (VTE), without a clear effect in mortality. 3 4 There is a limited understanding of which patients with CT have the highest likelihood of postthrombosis mortality. With the advent of alternative methods of anticoagulation with diverse bleeding and efficacy profile, 5 6 improved definition of the patients at risk is needed to better allocate preventive and therapeutic strategies.

The currently available mortality prediction tools for patients with VTE have performance limitations among patients with CT. Thus, the Pulmonary Embolism Severity Index (PESI) is a validated score to predict pulmonary embolism (PE)–related mortality. However, the score is only valid for patients with PE; the performance in patients with cancer is modest (area under the curve [AUC], 0.7), and cancer as a single variable is very heavily weighted in the score, potentially limiting dispersion of the model. 7 8 In contrast, POMPE-C was derived from a population with PE and cancer; it has a better performance in external validation (AUC, 0.8), but is only specific to patients with cancer who present with PE. 9 10 11 These scores have a segmented and limited approach to mortality prediction in CT; the scores assume that only patients with PE will have a high mortality. Fatal PE alone does not appear to explain the high death rate after CT. 12 The likelihood of fatal PE among patients with a history of VTE and cancer was described in the Registro Informatizado de Enfermedad TromboEmbólica (RIETE) database and was 1.4% at 3 months. This estimate differs from the mortality rate in current studies on CT therapy, which was as high as about 40% at 6 months. 13 14 Therefore, patients without PE on presentation have a high mortality risk that is not stratified with the current tools.

We decided to analyze the RIETE database to identify the group of patients with cancer and thrombosis with the highest risk of death within 30 days of a thrombosis.

## Methods

### Inclusion Criteria and Baseline Variables

We included patients with CT from the RIETE database. RIETE (ClinicalTrials.gov identifier: NCT02832245) is a multicenter, ongoing, global observational registry. Since 2001, we have used RIETE to evaluate outcomes after acute venous thrombosis. Consecutive patients with acute deep vein thrombosis (DVT) or PE confirmed by objective testing (compression ultrasound or contrast venography for DVT; helical computed tomography or ventilation perfusion scan or angiography for PE) were included. Patients with incidental, or arm, or visceral thrombosis were not excluded. Only the patients with adjudicated follow-up data for at least 3 months are considered valid entries in the registry. Active cancer was defined in those patients who have been diagnosed with cancer less than 3 months before VTE, patients with metastatic disease, or those receiving therapy at the time of diagnosis. Skin malignancies including melanoma were excluded.

Cancer-related variables included cancer type, metastatic disease, chemotherapy, hormonal therapy, and radiation therapy at diagnosis. Immobility was defined as bed rest for more than 4 days in the last 2 months. PE on presentation and bilateral versus unilateral DVT were also documented. Recent surgery was recorded if there was a major intervention in the 2 months preceding VTE. We obtained laboratory values including platelet count, hemoglobin, white blood cell (WBC) count, and creatinine at diagnosis. Demographics and comorbidities known at the time of VTE diagnosis were also recorded and included history of cerebral ischemia, symptomatic peripheral artery disease, prior myocardial infarction, hypertension, chronic kidney disease, diabetes, and smoking history. Data were collected via electronic case form on all participating sites (see Appendix). The data quality was regularly monitored for accuracy. All patients (or their relatives) provided written or oral consent for participation in the registry, in accordance with local ethics committee requirements.

### Statistical Analysis

We divided 2:1 the database between a derivation and a validation cohort using simple random selection. To demonstrate homogeneity of the derivation and the validation cohorts, we used Student's *t* -test and the Mann–Whitney *U* test for continuous variables and chi-squared or Fisher's exact test for categorical variables.

We categorized continuous variables in quartiles unless otherwise suggested in literature. To identify risk groups in the derivation cohort, we used a recursive partitioning and amalgamation method using a graphical approach to prune unnecessary splits. 15 16 We chose this strategy predominantly as a pragmatic approach to minimize the effect of multiple statistical interactions in this population, a “small *n* large *p* ” problem. 17 This is illustrated by over 1,000 potential combinations by analyzing only basic interactions in this database. In addition, recursive partitioning has been successfully used in multiple cancer and vascular thrombosis cohorts to define risk groups. 18 19 20 Finally, recursive partitioning is a valid alternative to Cox's proportional hazard for the analysis that obviates the assumptions of a constant relative risk through the follow-up. 21 After the selection of the groups of risk, we amalgamated two populations with the smallest dissimilarity. 22

The treatment received by the patients was not included in the models. We chose not to do so because of multiple reasons: during the first 30 days, the initial use of LMWH is a globally accepted standard of care; our goal was to define the death risk with descriptors available at diagnosis; and given that the treatment choices including filter placement or intensity of anticoagulant agents in the database reflect the real-life management of the disease that, in the absence of any, were not guided by a valid CT-specific mortality stratification score. There was no imputation of missing values.

We tested the findings in the validation cohort calculating the risk of death in each group and measured *p* -values for trend using a Cochran–Armitage test. To explore how robust were our findings, we isolated the analysis into predefined denominators: lung cancer, gastrointestinal malignancies, breast cancer, males. In addition, adjusted odds ratios (ORs) were calculated by controlling for relevant covariates by means of multiple logistic regression analysis. Accounting for multiple comparisons, we used a Bonferroni correction to a *p* < 0.002 as significant difference. Statistical analysis was done with IBM SPSS statistical program version 23 (Armonk, New York, United States).

## Results

We studied a total 10,025 patients with CT. The most common type of cancer was lung with 16.4% ( *n* = 1,658), followed by breast ( *n* = 1,418) and colorectal ( *n* = 1,392) with 14%. The median age was 69 years (range, 14–101 years), and most had metastatic disease ( *n* = 6,361, 63%). The derivation cohorts included 6,660 patients and there were no significant differences compared with the validation cohort ( *n* = 3,365) ( Table 1 ). Most of the patients in the derivation ( *n* = 6,153, 92.4%) and validation cohorts ( *n* = 3,089, 91.8%) received LMWH initially. There were 1,276 (12.6%) patients who died within the first month, evenly distributed between the derivation and validation cohorts.

**Table 1:** Baseline characteristics of the cohort

Variables	Cohort ( *N* = 10,025)		Validation ( *N* = 3,365)		Derivation ( *N* = 6,660)		*p-* Value
	n	%	n	%	n	%	
**Patient-related variables**							
Age (quartiles), y							
0–59	2,720	26.9	898	26.7	1,793	26.9	0.754
60–69	2,636	26.1	859	25.5	1,754	26.3	
70–77	2,503	24.8	851	25.3	1,635	24.5	
> 77	2,252	22.3	757	22.5	1,478	22.2	
Male gender	5,389	53.3	1,789	53.2	3,554	53.4	0.851
BMI							
Underweight	213	2.1	73	2.2	140	2.1	0.579
Normal	2,631	26.0	842	25.0	1,768	26.5	
Overweight	2,744	27.1	931	27.7	1,802	27.1	
Obese class I	1,090	10.8	362	10.8	711	10.7	
Obese class II	289	2.9	95	2.8	191	2.9	
Morbid obese	146	1.4	41	1.2	104	1.6	
**Cancer variables**							
Site							
Oropharynx/larynx	170	1.7	61	1.8	109	1.6	0.579
Esophagus	109	1.1	35	1.0	74	1.1	
Lung	1,658	16.4	569	16.9	1,089	16.4	
Breast	1,418	14.0	484	14.4	934	14.0	
Stomach	400	4.0	142	4.2	258	3.9	
Pancreas	509	5.0	180	5.3	329	4.9	
Colorectal	1,392	13.8	462	13.7	930	14.0	
Ovary	338	3.3	109	3.2	229	3.4	
Bladder	491	4.9	159	4.7	332	5.0	
Prostate	950	9.4	325	9.7	625	9.4	
Brain	332	3.3	104	3.1	228	3.4	
Hematological	735	7.3	220	6.5	515	7.7	
Unknown origin	255	2.5	90	2.7	165	2.5	
Uterus	377	3.7	109	3.2	268	4.0	
Kidney	168	1.7	52	1.5	116	1.7	
Others	497	4.9	181	5.4	316	4.7	
HCC	52	0.5	19	0.6	33	0.5	
Biliary system	138	1.4	50	1.5	88	1.3	
Vulva	36	0.4	14	0.4	22	0.3	
Metastasis	6,361	62.9	2,139	63.6	4,147	62.3	0.189
**Comorbidities**							
Bleeding in the past month	251	2.5	77	2.3	171	2.6	0.395
Prior myocardial infarction	216	2.1	138	4.1	272	4.1	0.528
Prior TIA or stroke	317	3.1	112	3.3	203	3.0	0.486
PAD	216	2.1	84	2.5	132	2.0	0.0129
Current smoker	774	7.7	273	8.1	500	7.5	0.326
Diabetes	1,065	10.5	342	10.2	709	10.6	0.642
Hypertension	2,682	26.5	912	27.1	1,741	26.1	0.165
Prior VTE	1,247	12.3	408	12.1	835	12.5	0.554
Immobility	1,935	19.1	665	19.8	1,246	18.7	0.205
Severe COPD	43	0.4	11	0.3	32	0.5	0.266
CKD	1,039	10.3	353	10.5	678	10.2	0.773
Surgery	1,037	10.3	367	10.9	670	10.1	0.189
**Medications**							
Statin	1,134	11.2	385	11.4	733	11.0	0.335
Chemotherapy	6,050	59.8	1,991	59.2	4,006	60.2	0.217
**Laboratory values**							
	**Median**	**IQR**	**Median**	**IQR**	**Median**	**IQR**	
Hemoglobin (g/dL)	11.79	2.12	11.75	1.96	11.82	2.20	0.384 a
White blood cells (×1,000/mm ^3^ )	8.80	5.30	8.42	4.66	9.11	5.48	0.343 b
Platelets (×1,000/mm ^3^ )	230.50	130.00	233.50	118.38	228.50	136.00	0.189 b
Fibrinogen (mg/dL)	551.50	222.50	555.00	224.00	549.50	210.50	0.213 b
Total cholesterol (mg/dL)	178.50	61.87	174.76	66.75	179.50	60.43	0.743 b
HDL-cholesterol (mg/dL)	41.76	15.17	41.21	15.50	42.01	15.06	0.176 a
LDL-cholesterol (mg/dL)	103.90	50.85	104.50	52.08	103.90	52.75	0.317 b
Triglycerides (mg/dL)	136.00	80.00	129.50	77.13	144.12	80.00	0.056 b
Creatinine (mg/dL)	0.90	0.44	0.86	0.45	0.90	0.42	0.303 b
**Outcome**							
Death within 1 month	1,276	12.6	429	12.7	827	12.4	0.636

Abbreviations: BMI, body mass index; CKD, chronic kidney disease; COPD, chronic obstructive pulmonary disease; HCC, hepatocellular carcinoma; HDL, high-density lipoprotein; IQR, interquartile range; LDL, low-density lipoprotein; PAD, peripheral arterial disease; TIA, transient ischemic attack; VTE, venous thromboembolism.

Note: Immobility defined as ≥4 days of bed rest in the last 2 months. All *p* -values were calculated with chi-squared test unless otherwise specified.

a Expressed as mean and standard deviation, *t* -test.

b Mann–Whitney *U* test.

**Fig. 1:**
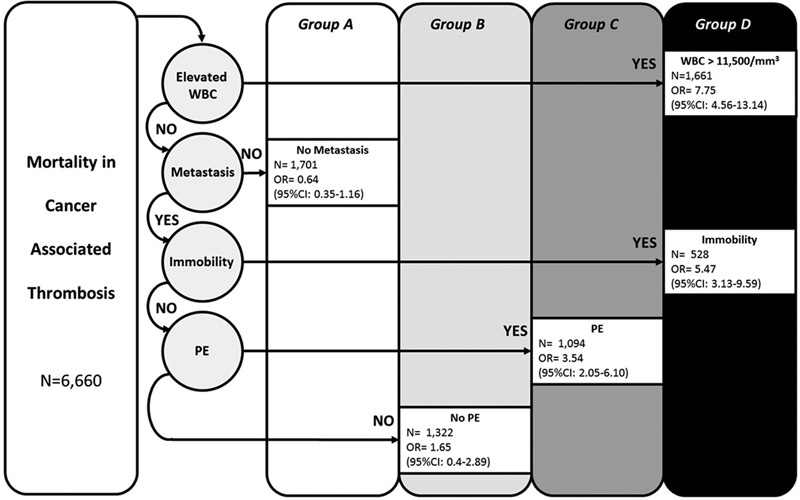
Groups at risk of early mortality after cancer-associated thrombosis in the derivation cohort. Derivation cohort death risk categories and odds of 30-day mortality. Patients with a missing variable were not carried forward in the count.

**Fig. 2:**
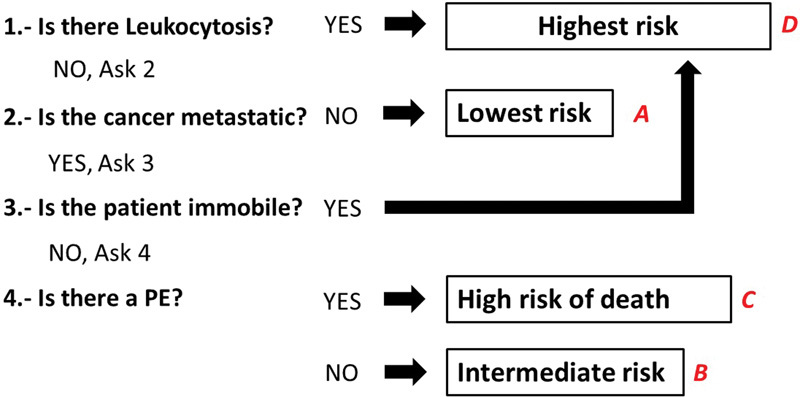
Sequence of questions to define risk groups.

In the recursive partitioning analysis of the derivation cohort, an increased WBC count level in the highest quartile was a strong predictor of early mortality (OR, 7.8, 95% confidence interval [CI], 4.6–13.1). In the recursive partition analysis, the presences of PE, metastatic disease, or recent immobility were the additional variables that created the regression tree for selection of the groups at risk. Increased WBC count was a better discriminator of death risk than the combination of metastatic disease, immobility, and PE ( Fig. 1 ). After controlling for potential confounders including chemotherapy, metastatic disease, PE at baseline, and age, the adjusted OR was 3.3 (95% CI, 2.9–3.8). Patients with elevated WBC count and the subset of patients with no WBC count elevation but presence of metastases and immobility had the smallest dissimilarity in the mortality risk prediction ( Fig. 1 ); thus, we amalgamated these groups into a single category. Therefore, the final risk groups were ( Fig. 2 ):

- Patients with high WBC count on diagnosis; or those with metastatic disease and immobility on diagnosis (group D, highest death risk).
- If there was no elevated WBC count or immobility, those with metastasis and PE were group C (high death risk).
- Patients without elevated WBC count, PE, or recent immobility but with evidence of metastases were group B (intermediate death risk).
- Patients with normal WBC count and no metastases were group A (lower death risk), regardless of PE or immobility status.

**Fig. 3:**
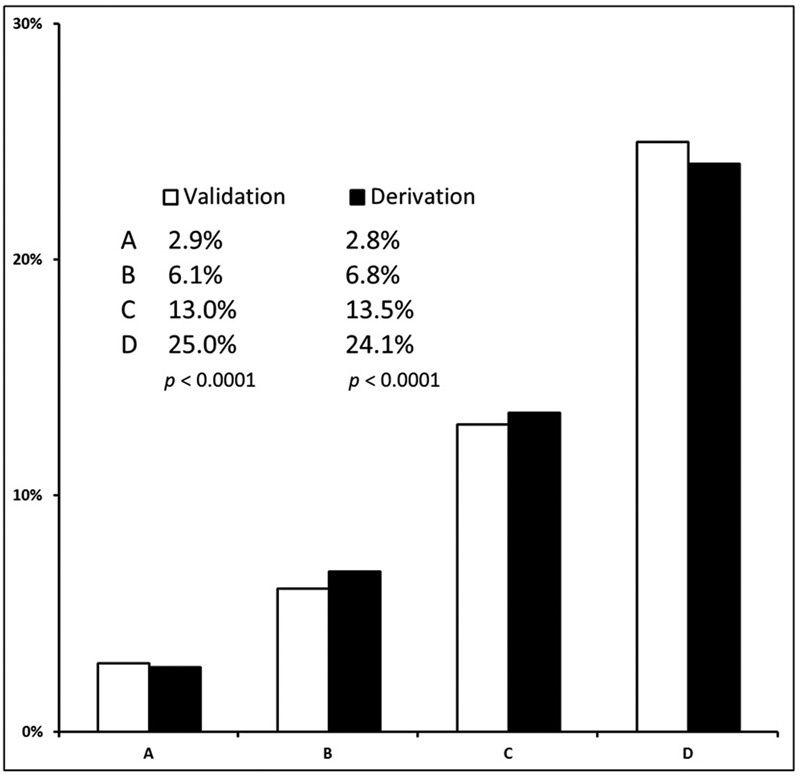
Trends for early mortality after cancer-associated thrombosis in derivation and validation cohort. Cochran–Armitage *p* for trend in early mortality after CT for derivation and validation cohorts.

**Fig. 4:**
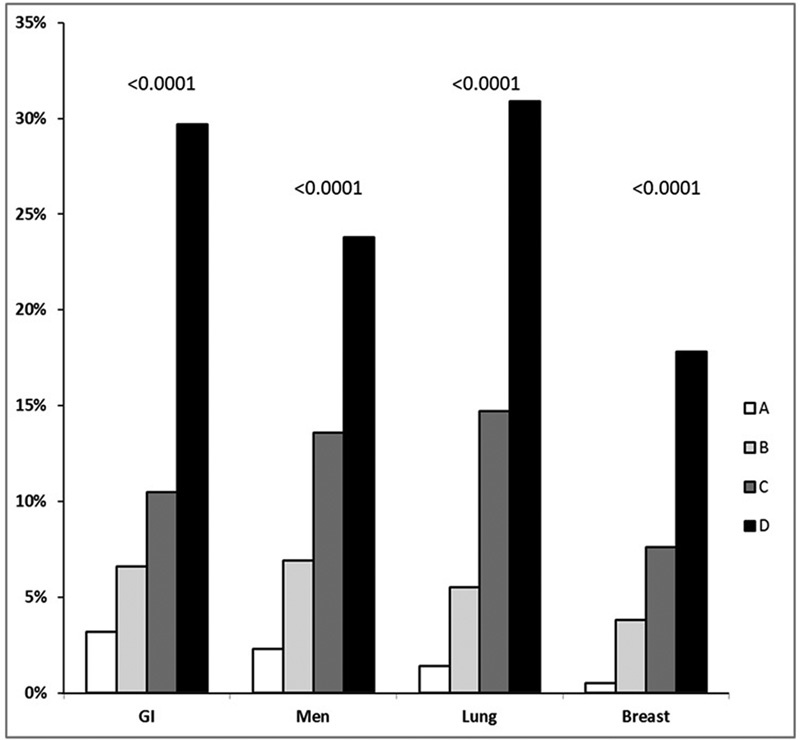
Early mortality by groups at risk in the validation cohort of patients with cancer-associated thrombosis. ( **A** ) No metastatic disease and no elevated WBC count. ( **B** ) Metastatic disease without PE, immobility, elevated WBC count. ( **C** ) Metastasis and PE without elevated WBC count or immobility. ( **D** ) Elevated WBC count *or* metastatic disease with immobility in patients without elevated WBC count. GI, gastrointestinal.

There were 429 (12.7%) out of 3,365 patients who died within 1 month in the validation cohort. We observed again an escalation on the risk of mortality within the first month according to the risk groups. The risk of early death after CT was 2.9% in group A (95% CI, 1.9–4.3), compared with 25% in the group D (95% CI, 22.5–27.5) ( Fig. 3 ). In the subgroup analysis, we found a significant *p* for trend among men ( *p* < 0.0001), women ( *p* < 0.0001) (not shown in the figure), and in patients with breast cancer ( *p* < 0.0001), gastrointestinal cancer ( *p* < 0.0001), and lung cancer ( *p* < 0.0001) only subsets ( Fig. 4 ).

We measured the main differences between those patients with increased WBC count and the rest of the cohort. Patients with elevated WBC count also had higher platelet count (236.00 ± 132 vs. 229.00 ± 131 × 10 ^9^ /L; *p* < 0.0001) and were less commonly receiving chemotherapy (47.8 vs. 63.9%; *p* < 0.0001), and most had metastatic disease (72.7 vs. 59.3%; *p* < 0.0001).

## Discussion

In a very large database of patients with CT, we have defined and validated groups of patients with escalating likelihood of mortality. We propose a simplified set of questions to rapidly categorize risk groups on presentation ( Fig. 2 ). Patients with elevated WBC count on diagnosis of CT, along with those with normal WBC count but with metastatic disease and immobility, had the highest 30-day mortality.

The reported mortality rate, demographics, and cancer type distribution in our study are consistent with that observed in recent cancer thrombosis trials. 6 10 11 13 In the EPIPHANY trial, it was already noted that among patients with cancer-associated PE, there were some predictors of death that overlap with our findings. 23 The authors evaluated 1,033 patients with PE and found that performance status and metastatic disease were predictors of death. Direct comparisons with our database are limited as patients with DVT only were not included. The Khorana risk assessment score, initially developed to predict CT among patients receiving chemotherapy, includes elevated WBC count within the classification. 24 The Khorana score has been associated with higher likelihood of mortality in patients with cancer, but in our analysis none of the other variables within the score predicted 30-day mortality. 25 26 Our findings add to a recent publication in which the value of the Khorana score as a mortality predictor in patients with CT had limited performance (AUC, 0.54; 95% CI, 0.52–0.56). 27 Only 23% of the fatalities were among patients with high-risk Khorana score. In contrast, in our current study, WBC count alone selects 50% of the early fatalities.

Strengths of our research include the utilization of a very large prospectively collected database, which has allowed us to derive and validate our findings; our findings were robust, and confirmed in several subsets of patients. A prediction tool, beyond its statistical performance, will only be as clinically useful as its ease of implementation. Our methodology allows for a simplified set of questions to rapidly estimate mortality risk ( Fig. 2 ). Moreover, the global nature of this database allows us to represent the likelihood of early mortality after CT with real-life generalizability. There are limitations to our analysis; there was no standardization of initial anticoagulant therapy. However, most patients with CT in the RIETE database are indeed treated with LMWH initially, as it is the current standard of care. 27 Among patients with PE, an increased right ventricle /left ventricle ratio seen in the CT would select patients with higher mortality risk. 28 We did not have access to this information; this limitation, however, is not pertinent for those patients who present without PE, and the role of the ratio in mortality prediction has not been definitively analyzed among patients with cancer. We did not have enough granular information as to explore potential medications and type of chemotherapy that could have explained our findings. The database does not uniformly contain troponin levels, which are an indicator of mortality among patients with PE. 29 Although the performance status was not prospectively entered, the value of immobility reflects on the known risk of death for patients with high ECOG status. We did not analyze stage other than metastatic versus not metastatic. The analysis of stage, however, is more likely to be valuable in single cancer–type models since equal cancer stage does not correlate with death risk across cancer types.

It was intriguing that WBC results predicted such a large number of patients with death. When we defined those patients with elevated WBC count, patients with metastasis and chemotherapy were more likely to have an increased WBC counts; but neither of those variables had a better discriminatory power than WBC alone. It is plausible that some of them were receiving steroids, which was not a collected variable. We do not know which patients had infections detected on admission; thus, sepsis may also account for some of the results. Elevated leukocyte count has already been associated with cancer-associated mortality in prior studies, but not deeply explored in the context of CT. 30 31 32 While chronic inflammation has been suggested as a link between mortality and malignancy, it is also associated with VTE incidence. 33 The degree of inflammatory response is another potential explanation of the observed death rate. The likelihood of death among patients without metastasis and who are mobile and do not have PE is low. Our findings may cause one to rethink the intensity of anticoagulation in this subset of patients since they have a very high likelihood of bleeding during anticoagulation.

In conclusion, we have derived and validated a robust set of characteristics that select patients with highest likelihood of death after CT. We present our result as an easy to implement, short sequence of questions. Our findings may be considered when analyzing risk and benefits of anticoagulation therapy, and may help adjust resource allocation with the growing number of therapeutic strategies in CT.

## Appendix

**Members of the RIETE Group:** SPAIN: Adarraga MD, Aibar MA, Alfonso M, Arcelus JI, Ballaz A, Baños P, Barba R, Barrón M, Barrón-Andrés B, Bascuñana J, Blanco-Molina A, Camón AM, Cruz AJ, de Miguel J, del Pozo R, del Toro J, Díaz-Pedroche MC, Díaz-Peromingo JA, Falgá C, Fernández-Aracil C, Fernández-Capitán C, Fernández-Muixi J, Fidalgo MA, Font C, Font L, Furest I, García MA, García-Bragado F, García-Morillo M, García-Raso A, Gavín O, Gaya-Manso I, Gómez C, Gómez V, González J, Grau E, Guijarro R, Gutiérrez J, Hernández-Blasco L, Hernando E, Jara-Palomares L, Jaras MJ, Jiménez D, Jiménez R, Jiménez S, Joya MD, Lecumberri R, Lima J, Llamas P, Lobo JL, López-Jiménez L, López-Reyes R, López-Sáez JB, Lorente MA, Lorenzo A, Loring M, Madridano O, Maestre A, Manrique-Abos I, Marchena PJ, Martín-Asenjo M, Martín M, Martín-Martos F, Monreal M, Morales MV, Muñoz C, Nieto JA, Nieto S, Núñez MJ, Olivares MC, Otalora S, Otero R, Pedrajas JM, Pellejero G, Pérez-Ductor C, Peris ML, Pons I, Porras JA, Ramírez L, Riera-Mestre A, Rivas A, Rodríguez-Dávila MA, Rosa V, Rubio CM, Ruiz-Artacho P, Sahuquillo JC, Sala-Sainz MC, Sampériz A, Sánchez-Martínez R, Sancho T, Soler S, Soto MJ, Suriñach JM, Tolosa C, Torres MI, Trujillo-Santos J, Uresandi F, Usandizaga E, Valero B, Valle R, Vela J, Vidal G, Villalobos A, Xifre B; BELGIUM: Vanassche T, Vandenbriele C, Verhamme P; BRAZIL: Yoo HHB; CANADA: Wells P; CZECH REPUBLIC: Hirmerova J, Malý R; ECUADOR: del Pozo G, Salgado E, Sánchez GT; FRANCE: Benzidia I, Bertoletti L, Bura-Riviere A, Falvo N, Farge-Bancel D, Hij A, Merah A, Mahé I, Moustafa F, Quere I; ISRAEL: Braester A, Brenner B, Ellis M, Tzoran I; ITALY: Antonucci G, Bilora F, Bortoluzzi C, Brandolin B, Bucherini E, Camerota A, Cattabiani C, Ciammaichella M, Dentali F, Di Micco P, Duce R, Giorgi-Pierfranceschi M, Grandone E, Imbalzano E, Lessiani G, Maida R, Marampon F, Mastroiacovo D, Pace F, Pesavento R, Poggio R, Prandoni P, Quintavalla R, Rocci A, Siniscalchi C, Tiraferri E, Tonello D, Visonà A; LATVIA: Gibietis V, Skride A, Vitola B; REPUBLIC OF MACEDONIA: Zdraveska M; SWITZERLAND: Bounameaux H, Mazzolai L; USA: Caprini JA.

**Coordinator of the RIETE Registry:** Dr. Manuel Monreal (Spain).

**RIETE Steering Committee Members:** Dr. Hervè Decousus (France); Dr. Paolo Prandoni (Italy); Dr. Benjamin Brenner (Israel).

**RIETE National Coordinators:** Dr. Raquel Barba (Spain); Dr. Pierpaolo Di Micco (Italy); Dr. Laurent Bertoletti (France); Dr. Inna Tzoran (Israel); Dr. Abilio Reis (Portugal); Dr. Henri Bounameaux (Switzerland); Dr. Radovan Malý (Czech Republic); Dr. Philip Wells (Canada); Dr. Peter Verhamme (Belgium); Dr. Joseph A. Caprini (USA).

**RIETE Registry Coordinating Center:** S & H Medical Science Service.
